# Book Reviews

**Published:** 1826-03

**Authors:** 


### CRITICAL ANALYSIS

OF

### ENGLISH AND FOREIGN LITERATURE,

RELATIVE TO THE VARIOUS BRANCHES OF

### Medical Science.

Quæ laudanda forent, et quæ culpanda, vicissim

Illa, prius, cretâ; mox hæc, carbone, *notamus.*—PERSIUS.

### DIVISION I.

### ENGLISH.

ART. I.—*A Practical Treatise on Diabetes: with Observations on the Tabes Diuretica, or Urinary Consumption, especially as it occurs in Children; and on Urinary Fluxes in general. With an Appendix of Dissections and Cases, illustrative of a successful Mode of Treatment; and a Postscript of practical Directions for examining the Urine in these Diseases.* By ROBERT VENABLES, M.B. Physician to the Henley Dispensary, &c. &c. &c.—8vo. pp. 214. London: T. and G. Underwood. 1825.

FOR what purpose this book was written, we cannot divine, unless it was to illustrate the truth of the old adage—that there is nothing new under the sun. It is the last work upon the subject, and, in our opinion, decidedly the worst. Our situation as reviewers condemns us to many a wearisome and unprofitable task: we are condemned to make many a fruitless plunge, ere we discover the pearl of which we are in search. It is not, therefore, to be wondered at if we are occasionally a little out of humour when our toils meet not with an adequate reward. Critics are generally considered a surly, ill-natured tribe, who will not be pleased. Now, though we do not at all affect the cynic, yet an involuntary exclamation of impatience will escape us when we are unnecessarily intruded upon. He who writes a book either actually has, or pretends to have, information to communicate, which they to whom it is addressed do not possess. He who actually possesses superior knowledge, and imparts it, is a public benefactor: he who only pretends to it, voluntarily exposes himself to criticism and to censure.

In his Preface, Dr. VENABLES informs us that two causes induced him to write this book,—viz. that he had discovered, first, that an excessive discharge of urine is frequently a cause of tabes in children; secondly, that phosphate of iron proves, when properly administered, almost as certain an astringent with regard to the excessive action of the kidneys, as opium in that of the alimentary canal. Of such importance did these two facts appear to the mind of Dr. Venables, that he seems to have thought any other mode of communicating them to the profession, than in a large octavo, as too obscure and insignificant. That, however, he should have hid these two grains of wheat in two bushels of chaff, we do sincerely regret, for he is evidently an industrious man. We do not wish to damp his zeal; but, if he would follow the precept of the poet,—he knows to what we allude,—and not be quite so precipitate and hasty in his productions, we might expect from such industry something useful to the profession, and honourable to himself. As it is, this work will not be read (except by the few who, like us, are doomed to the task of reading every thing,) until PROUT and his fellow labourers are forgotten.

The first part of his book is taken up with observations on Tabes Diuretica, as it occurs in children.

"There is a cause of emaciation among children, which has hitherto attracted but little of the attention of the profession. I have often observed children to all appearance very healthy up to a certain period, when suddenly the constitution changes, the child emaciates, its health declines, and, without any obvious derangement sufficient to account for the gradual depravation of health, at last dies a most miserable object. In such cases, the head, chest, and abdomen, present no morbid appearances sufficient to account for the wasting and gradual decline of health. Accident led me to a discovery of the real seat of disease in such cases; and, when the history of the complaint has been submitted to the reader, he will not be surprised that its nature and seat should have so long escaped general observation. (P. 1.)

The nature and seat of this disease has not, as Dr. V. would wish us to believe, escaped general observation. Dr. UNDERWOOD, in his work on the Diseases of Children, has devoted a chapter to Polydipsia, and another to Diabetes infantilis. In the former he mentions that ZUINGERUS speaks of immoderate thirst, accompanied with a great flow of urine, as a common affection among young children; and he adds, that in a little time their bellies become tumid, that they are subject to glandular affections, and fall into atrophy. This description seems to bear some resemblance to the tabes diuretica of Dr. Venables. MORETON, also, in his Phthisiologia, mentions consumption from diabetes as a common disorder among children. These writers have, then, very ill-naturedly anticipated Dr. Venables in his discovery; and this last new fact proves, like many others, to have been just as well known to our grandsires as to ourselves. That diabetes is a disease from which no age or sex can plead exemption, has long been known to every physician at all acquainted with his profession; so that for Dr. Venables to produce this as a new fact, either evinces ignorance on his own part, or a very mean opinion of the attainments of his professional brethren, which it is our duty to convince him that they do not deserve.

Dr. Venables tells us that it was by mere accident that he stumbled on this fact—a child was presented to him for examination. "The head was free from pain, the functions of the brain regular, the respiration natural, the bowels free, and the secretions from them healthy." Nevertheless, the child was greatly emaciated. The Doctor was sorely perplexed to divine the cause of this. What! could it be possible that every function should be regular, and all the secretions and excretions natural, and yet that the child should be rapidly wasting away? He determined, therefore, to cross-examine, and at length discovered that the urine was discharged in great abundance. The enigma was solved,—the merit of Œdipus was his, and "he had no longer any hesitation in referring those cases of emaciation, which he had previously considered as anomalous and unintelligible, to this genus."

Now, such is the most unfortunate constitution of our mind, that, in spite of the positive assertion of our discoverer to the contrary, we cannot bring ourselves to believe that diabetes is so frequent a disease as he represents it to be. That more children may die of this disease than we are aware of, is very possible, and is a proposition which we feel no inclination to controvert. But we do not, and cannot, believe in its frequent occurrence; or that a large proportion of those who are treated for hydrocephalus and mesenteric affections, rickets, and all the other species of scrofula, are really afflicted with this disease. Nor do we speak on this subject without having en[illegible]ed extensive opportunities of witnessing the diseases of early life. The date of the earliest case recorded in the Doctor's book is in the year 1818. Of these cases there are only eleven. The author has nowhere asserted that he has seen more, and he has not furnished us with any table by which we can judge of the comparative frequency or infrequency of their occurrence; so that, though the author, as physician to a public dispensary, was happily situated for the discovery of such cases, did they frequently occur, he has only been enabled to collect, since 1818, eleven cases; and the greater portion of these were not true diabetes. This is by no means a number that entitles him to call it a disease of frequent occurrence.

The first chapter contains an enumeration of the symptoms:

"The disease seldom, if ever, appears till after the child has been weaned. The reason of this, perhaps, is, that the exciting causes are seldom applied till after this period. A child which has continued healthy up to this time, will perhaps suddenly lose its usual flow of spirits, become dull and inactive, and, although no obvious disease may be recognisable, yet the child will not appear in its usual health. It begins, after a very little time, to waste in flesh, and then gradually continues to emaciate. The skin becomes harsh, dry, and flabby, and seems to hang loosely about the body. The temperature is generally very much elevated, and, in the description of nurses, it will be said that 'they burn like a coal of fire.'" (P. 10.)

In the early stages, the bowels are said to be regular, and the appearance of the alvine discharges is natural and healthy. The tongue does not give any indication of disease, until the patient becomes hectic, and then it is covered with a coat of mucus. After the disease has continued some time, the bowels become irregular, and the fæces are sometimes of a greenish hue; at other times they are natural when evacuated, but assume a greenish tinge on standing. At a more advanced period, the abdomen seems preternaturally full and distended; and the pulse, from the first, is accelerated, and has a hard wiry feel. "The most remarkable symptom" (remarkable, we presume, from not being remarked, for our author assures us that it is generally overlooked,) "is the inordinate discharge of urine." This is accompanied with great thirst; but the Doctor asserts that the quantity of urine greatly exceeds the whole of the aliment, both solid and fluid. This is an assertion to which we can scarcely assent, unless the author is prepared to prove that the body possesses some hygrometrical properties, and can imbibe moisture like a hair. There are cases of diabetes recorded, in which the quantity of urine did not at all exceed the natural proportion. There are others in which the quantity discharged was certainly enormous. CARDANUS describes the case of a girl, who passed thirty-six pints per diem, when her body weighed only 260 lbs. and her meat and drink only 7 lbs; and SCHENCKIUS mentions the case of a woman, who passed in a few days a quantity of urine, that outweighed her whole body. Were it not that these things do most undoubtedly appear in print, we should be inclined to disbelieve them. We fear, however, that we must intimate our assent to the opinions of Dr. Prout, who says that, in the "best authenticated cases," this enormous difference between the quantity of ingesta and urine has not been observed; and of Dr. Watt, who says that, in all the cases he had seen, the urine was less than the ingesta. "For a night or a day, or even a longer period, the urine might exceed what was taken in; but, on a more extensive average, it has always come short."

The urine is sometimes limpid, at others milky; sometimes of a straw colour, at others of a greenish hue. It is sometimes albuminous, and coagulates on the application of heat; and its specific gravity is increased. The emaciation is slow or rapid, according to the greater or less quantity of coagulable matter which it contains. Sometimes it is tinged with blood, with mucus, or with pus. The most remarkable thing in the urine of diabetic patients, is the large quantity of saccharine matter that it contains.—It is useless to point out the identity of this description with that given by Dr. Prout.

Dr. Venables does not think the division into the insipid, and what he calls the "mellitic" form of the disease, is well founded. We shall not stop to argue this point. His own cases, however, prove that there is a disease, characterised by a large flow of urine, by great emaciation, inextinguishable thirst, and voracious appetite, in which the urine is not at all saccharine.

As the disease advances, the head becomes affected, and we have headache, vertigo, and delirium. The patient often dies comatose, sometimes apoplectic. The organs of sense, (we follow closely the arrangement of the author,) also become affected, and the patient often complains of unpleasant odours, bad tastes, and dimness of sight. His skin is dry and harsh, and hectic fever comes on, with profuse nocturnal sweats.

The disease frequently terminates in anasarca; ascites sometimes supervenes in adults, rarely in children. The enlargement of the abdomen in children may, he says, be frequently mistaken by a careless observer for ascites, or some mesenteric affection; which latter affection he regards as an adventitious, rather than an essential occurrence. Pulmonic affections he also considers as of a secondary and symptomatic nature. To us both of these opinions appear to have been hastily adopted, and without sufficient consideration. There can be no doubt that diabetes can exist without disease of the lungs, but it is equally certain that it is very often complicated with such disease; and, as we know as yet nothing clearly of the nature of diabetes, it is, we think, a little precipitate to exclude antecedents such as these, which have so powerful an influence on the general health, from the class of what are called in medical language *causes*. Indeed, Dr. Venables does afterwards enumerate among the causes of the disease whatever is capable of producing general debility. In the same precipitate manner, he concludes that the derangement of the digestive organs, which he admits to be a very prominent symptom, is a consequence rather than a cause.

To refer every thing to the kidneys, and to exclude from our attention every other portion of the economy, is to take a too narrow and confined a view of the subject. Dr. Prout has taken, in the following remarks, a view of the question more consonant to truth:—"No one will assert that the kidneys can form lithic acid from any substance indiscriminately presented to them; they must have the ingredients on which they operate prepared in an uniform manner, and a series of preliminary operations must take place, every one of which must be presumed to be perfect, before the kidneys can be presumed capable of performing their duty correctly. The chief of these preliminary operations are digestion and assimilation; and hence it becomes evident that, if these important processes are in any way deranged, those of the kidney will be more or less affected."

We next proceed to the *morbid anatomy* of diabetes. Here again our author dismisses from his consideration every other organ but the kidney, and those immediately connected with it. The Doctor, however, has not, in our opinion, succeeded in establishing his favourite position—that disease of the kidney is the immediate cause of diabetes. The appearances which dissection has disclosed, have been very various; but the most common occurrence is that of enlargement of the kidney, and a gorged condition of its vessels. Sometimes the excitement has proceeded to inflammation, and "a whitish matter, resembling pus," has been found. Occasionally an abscess has been discovered; and Dr. Venables has seen two cases in which the kidneys were ulcerated. The ureters have been found more capacious than usual, and the renal arteries enlarged. Disease of the bladder has also occasionally occurred.—Such are the usual and unusual appearances of these organs. Those of most frequent occurrence are such as indicate a high degree of excitement, rather than a "very diseased state of the kidneys."

As to the other viscera, whatever morbid appearances they exhibit, they are to be forgotten entirely in considering the morbid anatomy of this disease, because Dr. Venables considers them rather as accidental occurrences, than as having any essential connexion with the disease. Graver authorities have, however, thought otherwise. Dr. WILLAN observes, that he never saw a case of confirmed diabetes, wherein there was not some considerable disorder of the constitution, or a defect of some organ essential to life.

The third chapter treats of the *causes.* In this category all diuretics are classed,—all spirituous and fermented liquors, acids, alkalies, and alkaline salts. Gin especially comes under his ban. Hard and sour ale, sour milk,—foreign, acescent, and home-made wines. These things may have a remote effect in producing the disease, for aught that we know; but we are inclined to think that it is a very remote effect indeed. The disease has always been a rare one. CULLEN saw only twenty instances of it; HEBERDEN witnessed the same number. Considering the extensive practice of these great physicians, this number is very small. It ought, however, to be very frequent, if such causes had any decided and powerful influence in its production. By intemperate indulgence in some of these things, the general health may be destroyed; and we are inclined to believe that this disease seldom, if ever, arises until the constitution has received a severe shock, and the whole system has become depraved and vitiated.

In common with other writers on this subject, Dr. Venables believes that the disease may be transmitted from father to son.

The immediate cause of the disease, is some undefined and undefinable condition of the kidneys. "Of what nature are these changes, or wherein their difference consists, we probably shall never be able to determine."

Having thus described the symptoms, the morbid anatomy, the causes remote and immediate, we arrive at the section devoted to the *pathology* of the disease. Pathology, we are told, is the doctrine of these morbid operations, by which morbid effects are produced. From this definition, we expected to find some new information on this difficult part of the subject; but we are merely told, that the author can no more account for the secretion of sugar, than why the kidneys secrete urine instead of bile. Instead of throwing any light upon the nature of the disease, we have again to travel over the same ground we have just past, and commence a new disquisition upon the causes of the disease, which we are again told are gin, brandy, acids, alkalies, diuretics, *et hoc genus omne*; and that, as these are the causes, diabetes arises from a "peculiar excitement of the kidneys."

It is well known that many substances are no sooner introduced into the system, than they are absorbed, and may be found mixed with the urine. These substances, we are informed, are accommodated with a more direct path to their destination than the long and circuitous route of the circulation. Of the "highways" of the body, we do flatter ourselves that we know something; of the "byways," nothing; and we confess that we know of no other means by which any thing absorbed can arrive at the kidneys, than through the medium of the arteries.

The voracious appetite, excessive thirst, and dry harsh skin, are easily accounted for. We are again told that all the other diseases of the viscera are secondary: here, however, he does qualify and limit his assertion, and admit that diabetes may be a secondary affection.

Chapter the 4th is on the *diagnosis*, and contains merely a condensed enumeration of the symptoms.

The succeeding chapter is on the *prognosis*. In this we are informed, that the young are more easily cured than the old; that remedies are more efficacious in the incipient than con-

There needs no ghost to tell us that firmed state of the disease; and also that it is more difficult to cure when some serious disorganization of the kidneys has taken place;—that the longer the duration of the disease, the greater will be the disorganization of the kidneys, and the less will be our chance of removing the complaint, &c. The supervention of dropsy is to be regarded generally, though not invariably, a fatal indication. In one case, where "dreadful pains were felt in the loins, the spinal marrow in the lumbar region was found, upon dissection, very much diseased."

We advance, in the sixth chapter, to the *treatment* of diabetes. It is fortunate that, though we know little of the nature of the disease, we are possessed of a mode of treatment which has in many cases proved successful. The remedial means proposed by Dr. Venables do in no respect differ from those recommended by his predecessors. The exciting causes are, if possible, to be ascertained and removed. This alone will frequently be sufficient, in the early stage, to effect a cure. If, however, the excessive action of the kidneys continues, venesection should be employed. The experience of Dr. Venables as to the utility of this practice, is quite in accordance with that of Dr. Watt. The quantity of blood abstracted must depend upon circumstances; but he recommends us to have recourse to repeated venesections, rather than diminish the patient's strength by one large and copious detraction of blood. If there be pain in the loins, leeches, cupping glasses, and blisters, are recommended; and if, from the severity of these pains, we have any reason to apprehend disorganization of the kidneys and disease of the spinal marrow, caustic issues will prove of efficacy. The bowels should be kept open, and perspiration promoted. He also bears testimony to the power of opium in this disease, but advises its conjunction with an antimonial. In common with other writers, he advocates the exhibition of phosphoric salts, which he considers as having a powerful restringent influence upon the action of the kidneys. After asserting that phosphate of soda has such an effect, and adducing, in corroboration of his opinion, the testimony of Dr. SHARKEY, of Cork, he makes an observation, which appears rather contradictory of his previous assertion. He says that the alkalies and alkaline earths excite the kidneys, and that he has "his doubts" whether this property can be overcome by any combination.

However, the good effects which he expected from phosphate of soda were apt to be frustrated, "by its passing off by stool before its effects upon the kidney could be secured." This circumstance determined him to substitute the metallic phosphates for those with an alkaline base. The experiment succeeded beyond his expectations. "I have been really struck with the efficacy of the phosphate of iron, in excessive discharges of urine. The quantity is rapidly reduced under the use of this salt, and its qualities sensibly altered. The bulimia, which also attends on diabetes, is reduced, and the powers of digestion invigorated and increased." We cannot agree with Dr. Venables in attributing all these good effects solely to its astringent effects upon the kidneys. Indeed, we see no decisive proof of its having any peculiar and specific action on these organs. We can, however, readily believe that, along with other preparations of this metal, it may act as a tonic, and, by imparting vigour and energy to the whole system, may diminish the discharge, and promote the restoration of health.

It is now our duty to show that Dr. Venables is by no means the discoverer of this "new remedy," which is nothing but an old one brought forward anew. It is painful to us to be obliged to prove that his pretensions to this merit are groundless,—to subvert a reputation which he imagines himself to have firmly established; but justice demands this at our hands. Though he has failed here, a great field still remains open for his exertions. The map of medical science is but half unrolled; and even that portion is but ill filled up,—there are in it great voids and spaces; there is much terra incognita, much both to stimulate and reward industry. To this unoccupied ground we respectfully beg leave to direct his attention: his industry will reap a much richer reward from its cultivation, than from retailing at second-hand the opinions and practices of others.

If, then, previous to the composition of his Treatise, Dr. Venables had taken the trouble to read Dr. LATHAM's work upon Diabetes, he would have found the following remarks:—"For the purpose of restoring the vigour of the system, when the diabetic tendency has been checked, nothing would perhaps be better than iron combined with phosphoric acid, as it is found in the phosphat and oxyphosphat of that powerful mineral; and, when a purgative is wanted, soda phosphorata should always be preferred. In chronic diabetis, more especially, I have seen this plan very beneficially employed." The remarks of Dr. Venables are, however, deserving of attention, since his experience so fully coincides with that of Dr. Latham.

"The prophylactic treatment," we are told, "consists generally in increasing the strength, and promoting the healthy action of the different functions; and the proper regulation of diet, air, and exercise, and in shunning intemperance."

The Appendix consists of cases and dissections; and the volume is closed with a Postscript, containing directions for a practical examination of the urine, similar to those given by Dr. Prout and other writers, whose works are in the hands of the profession.

We have now given a fair account of the contents of this volume. We are not aware that we have omitted any thing of importance; we have suppressed nothing, distorted nothing. We have not condemned Dr. Venables unheard. We leave it to the profession to judge whether or not we have redeemed our pledge, and shown that he has constructed his work out of materials culled from the works of others. Those who have studied the writings of ROLLO, and WATTS, and PROUT, will find in this Practical Treatise no information which they did not previously possess. Those who take it up entirely ignorant of the subject will, of course, glean considerable information; but we should advise them to drink at the fountain-heads.

---

### DIVISION II.

### FOREIGN.

ART. II.—*Grundriss der Physiologie.* Von D. KARL ASMUND RUDOLPHI, Prof. d. Med. u. Mitgl. d. Königl. Akad. d. Wiss. Erster band.—8vo. pp. 297. Berlin, 1821.

*Elements of Physiology.* By K. A. RUDOLPHI, M.D. Translated from the German, by WILLIAM DUNBAR HOWE, M.D. Vol. i.—Pp. 254. London, 1825.

To those who are acquainted with the name of RUDOLPHI, merely as the author of a scientific work on the *Entozoa*, it may not be superfluous to mention that he occupies the chair of Anatomy in the University of Berlin. In this situation, physiology, as a matter of duty, must have engaged some share of his attention; but there are much more satisfactory proofs of his attachment to it as an object of study, and of the interest he takes in its advancement as a science, in the publications he has at different times given to the world, either in a separate form, or in the Transactions of the Academy of Sciences of Berlin.

Familiar with human anatomy, learned in comparative anatomy,—equally knowing in every other branch of natural history,—and possessing much acuteness and discrimination, he brings with him to the composition of a work on Physiology advantages not always possessed by those who have undertaken to present us with a summary of our knowledge on this extensive subject. We say extensive subject, because physiology, in the wide and proper acceptation of the term, embraces the organisation and functions of all living beings; and human physiology, which is but a branch of it, is never to be advanced to the rank of a natural science by the exclusive consideration of man, but must receive its most correct illustrations, and most solid support, from the investigation of the structure and functions of all living beings, from the anthropomorphous simiæ down to the simplest forms of animal and vegetable existence. Limited as our knowledge still is in this wide and almost boundless field of inquiry, it is from the constant reference to facts in general anatomy and general physiology, in aid of its more specific object, human physiology, that the work of Professor Rudolphi, which we are about to notice, derives its greatest merit and best claim to commendation. Indeed, we are inclined to regard it, upon the whole, as the best work on Physiology which has been published, from the comprehensiveness and general correctness of its views, the complete and condensed nature of its details, as well as from the number and value of the facts which it contains.

Such being our opinion of the original work, it is with regret that we find ourselves unable to mention in favourable terms the manner in which it has been presented to the English reader. The faults of the translation are such, as in many instances to misrepresent, and in numerous others to render imperfectly, the meaning of the author: but the nature and extent of the errors of this publication we shall subsequently take an opportunity of pointing out,—at present we recur to the original.

The Introduction, having given a definition of physiology, presents us with the division of the subject adopted in the succeeding work, with a brief sketch of the aids to be derived from the accessory sciences, natural history, natural philosophy, chemistry, anatomy, and pathology; and with a list of the literature of physiology, or of the works which have been published upon it in different European languages.

Physiology is defined to be the history of the *human organismus;* and, as this is a term which has been misunderstood, and may occur frequently in the course of our analysis, we add the succeeding remarks to elucidate its meaning. A living organismus is never spoken of,—an organismus without life being inconceivable; when the first is formed, the latter invariably attends it: at the same time, we cannot say that life produces the organismus, or that the former only ceases when the latter is destroyed; so that a human carcase, or an animal, preserved in spirits, is not an organismus, but merely a certain amount of its parts. The organismus is the source, not only of the corporeal, but of the mental powers.

Physiology divides itself into two parts, a general and particular: the former viewing the organismus as a whole; the latter entering into the consideration of its individual parts. General Physiology includes, *a*, Anthropology; *b*, Anthropotomy; *c*, Anthropochemistry; *d*, Zoonomy. Particular Physiology includes, *a*, Sensation; *b*, Motion; *c*, Nutrition; *d*, Generation.

Part I. section i. *Anthropology*, or the natural history of man, compares him with other animals, and thereby establishes his peculiar and distinctive characters, so as to assign him his rank amongst created beings; whilst it likewise compares, or contrasts with each other, the various tribes of people inhabiting the earth, in order to discover the circumstances in which they resemble or differ,—the affinity or diversity between them.

Chap. 1. *Difference between man and animals.*—Belonging to the class Mammalia, and in external form, as well as in internal structure, more closely approximating to the Quadrumana, the resemblance between man and the simiæ was formerly greatly exaggerated. This

6

arose, in a great measure, from the circumstance of the Pongo, when young, having a greater similarity to man, and having been, therefrom, described as a distinct species, under the name of orang outang. The observations, however, of Tilesius, Cuvier, Lawrence, and those of our author, would render it extremely probable that the young Pongo is the anthropomorphous animal, the orang outang, which would thus merely exhibit one of those intermediate stages of development, wherein animals, in individual parts, more nearly resemble man. In making comparisons, also, between man and animals, we must take the former in his perfect state, and not select individuals in a state of both physical and moral deformity, as is shown to have been the case with all those instances about which so much noise was formerly made as exhibiting the original type of man.

The numerous and important distinctions between man and animals have all reference, without exception, to his destiny to live as a rational being, whilst animals, merely influenced by sensual impulse, never rise to the formation of general ideas. On the other hand, several distinctions, formerly supposed to exist between man and animals, (for which even moral reasons were supposed to have been discovered,) have, on closer examination, been found not to hold good; such as the presence of the hymen, and the existence of the menstrual discharge.

The upright gait is natural to man alone of all the mammalia, that is, it is a natural consequence of his structure; so that it is universal, and exists even in tribes living in the lowest state of barbarism. The peculiarities of structure in the lower extremities, pelvis, spinal column, and chest, connected with this erect attitude, are indicated, as well as those of the superior extremities, of the head, the greater proportion of brain, the preponderance of the bones of the cranium over those of the face, the facial angle, &c. We find it remarked, that the pieces of bone which correspond to the ossa intermaxillaria of animals, sometimes remain distinct to the fourth month in the human embryo. Frequently a trace, or indication, of them is seen, in the form of a suture behind the incisor teeth; sometimes they are preternaturally developed, so as to form the double hare-lip, or rather double hare-lip fissure, in which case our author has hitherto seen but one incisor tooth on each side in the projecting pieces of bone. The naturally unarmed and defenceless state of man,—his prerogative of speech, facility of adaptation to existence in every climate, slow growth, and late puberty,—his intellectual and moral character,—and, lastly, the diseases which he has in common with other animals, and those which are peculiar to him, are successively brought forward.

Chap. 2. *Differences of mankind from each other*.—All the people of the earth coincide in the possession of those distinctions from animals which have been pointed out, and collectively constitute one genus; but they differ in the most manifold manner from each other, in stature, in the form of the body generally, and of its individual parts, more especially of the skull and of the face; in the texture and colour of the skin and hair; and perhaps also in perfectibility, which does not seem uniform in all the inhabitants of the earth.

Stature is one of the least constant, and therefore one of the least important, differences. It is, upon the whole, greatest among the inhabitants of the temperate zones, and least amongst those who live in high northern latitudes: the Tenuels or Patagonians, who attain the height of from six to seven feet, are the highest; and the Laplanders and Eskimaux, who seldom reach five feet, the lowest in size of human beings. The greater or less stature of whole nations will be found to depend on the greater or less development, longitudinally, of every part of the body; whilst, in the case of isolated tall individuals, it will be found connected with the development of a particular part, the spinal column or lower extremities.

The form of the body, no doubt, presents considerable differences amongst the individuals of the same nation; but it is unquestionable that, in certain original stocks of men, there prevails a predominating fineness of form, a greater degree of symmetry, and a more athletic make; from whence we descend, through numerous gradations, to the extreme of human deformity, in the Australian negro. It is in the form of the head, however, that the principal diversities exist among mankind; either all parts of the cranium, especially the forehead, being much developed, or the latter receding, and the sides of the cranium being compressed; whilst, on the other hand, the jaws and zygomatic arches either project or recede,—differences which do not occur gradually, but are evident even in the fœtus. The forms of the bones of the cranium, and of the face, have considerable influence on those of the soft parts,—as on the position of the eyes, the direction of the nose, form of the chin, &c.; whilst others depend on the soft parts themselves,—as the narrow aperture of the eyelids in the Mongols, the tumid lips of the Negro, &c.

The colour of the body is in some tribes white, in others brown, yellow, red, or black, with various gradations of each. It is mainly dependent on organisation, and not upon climate; the skin of the Negro, for instance, besides being black, has a peculiar softness and lubricity.

With the colour of the skin, there generally is a corresponding colour of the hair; and these are connected also, in most instances, with certain differences in quality. The yellowish blond hair of the northern Europeans is soft and fine, the dark and black hair of the southern, on the contrary, harder and coarser, yet that of the Hindoos is fine and long; the black hair of the Americans and Mongols is thick and rough, that of the Negro woolly and tufted. Besides quality, the hair differs also in quantity: its growth is greatest in the European stock, less so in the others. The little beard possessed by the American tribes is well known; and something similar is the case with the Mongols.

In muscular power, the Europeans exceed all others; whilst it is least in some Mongolian and Malay tribes.

But it is not only in corporeal qualities that the stocks of men differ; their intellectual powers do not appear to be the same in degree, and this is connected, apparently, with the greater or less development of brain. The case of an individual Mongol or Negro, under foreign guidance and instruction, accomplishing something, is no argument against the general inferiority of these stocks. A Negro has as much sense as an Irishman, at least.

Perhaps, even the parasitical animals of men differ. In the Russians and adjacent Prussians, and in the Swiss, we meet with the Bothriocephalus latus, (*tænia lata*, Linn.)—in the other Europeans, and in the Greeks, the *tænia solium*. Our author only knows of one case, that of a lady, (perhaps of mixed origin,) where both were met with. Of the intestinal worms of the Americans, Negros, &c. we know nothing.

The differences above enumerated sometimes occur singly, and are then of little consequence; but generally several of them are united in a certain combination, are permanent, and thereby form essential characters. The Negro is not only black, but the skin has a peculiar softness and a peculiar perspiration; his hair is woolly, his cranium compressed, and the forehead flat and receding; the jaws prominent, &c. The attempts, therefore, to account for these differences singly, avail nothing; for instance, taking the complexion or the features, and showing that in the same nation varieties of these occur: how they all occur combined, as in the Negro, is to be explained. The attempts alluded to have proceeded upon the assumption that all mankind have descended from a single pair, who had the European form; an assumption which, after some observations on the difficulty of its accounting for the present extent of the population of the earth, and the spread of this from a single point, our author declares to be in his opinion untenable, when we regard the differences among mankind. Uncontaminated races of men, even in the most opposite climates, have never altered their character: the Negro is the same now as in the remotest periods of history, and is the same in America as in Africa; the Jews and Gipseys still show their foreign origin. The produce of Europeans in other climates is never Negro nor Malay.

Although the different stocks of men breed with each other, this is no test of their common origin: how many similar animals and vegetables occur in different regions, without our having thereby cause to derive them from a single spot? What should hinder that, at different places, under the same conditions, they should originate? When we find in foreign animals, and in foreign climes, (for instance, in Brazil,) the same intestinal worms as in domestic animals, shall we derive them from one spot? In as little does the breeding of mankind among each other prove them of but one species; for, in the first place, even was it shown that all animals in a state of nature breed only with each other, this would prove nothing of man: and, in the next place, we hardly know of men in this wild state, and, where we do, they also restrict themselves to their own stock,—nay, even in civilised communities, this is generally the case. If our only test of species among animals was the proof of experience having shown that they do not breed together, we must, indeed, then enumerate but few; for, of how many have we ascertained this?

Upon the grounds, therefore, which guide us elsewhere in natural history, M. Rudolphi regards mankind as constituted of several species; and, on this account, he objects to the term race, or variety, for distinguishing the different stocks of men, proceeding, as it does, on the assumption, which he thinks cannot be established, of all being descended from common parents. Several of these distinctions are so great and so permanent, that it were to be wished we had marks equally characteristic with regard to other animals. The principal indicate to us the European stock, the Mongolian, the American, and the Negro. BLUMENBACH assumes five; but our author thinks the Malay a mixed race. The characteristics of these different stocks we need not go through: we may only remark that, in opposition to the celebrated naturalist just mentioned, our author ranks the Laplanders, Finlanders, Eskimaux, and Tschutski, as belonging to the European or Caucasian stock; and this from personal observation of individuals of these different tribes. Blumenbach had referred them to the Mongolian.

Section II. *General Anthropotomy.*—After pointing out VESALIUS, FALLOPIUS, HALLER, and SOEMMERING, as having paid some attention to parts of this subject, but that to BICHAT belongs the merit of having subjected to multiplied experiment and observation the various tissues which, either upon anatomical or physiological grounds, he considered elementary, and having thereby almost formed a new science, general anatomy,—our author proceeds, in Chap. 1, to consider the *simple solids of the body.* Bichat's division of these into twenty-two systems, is objected to as being physiological, rather than anatomical; and the following are adopted as the elementary solids:—Cellular tissue, horny tissue, cartilaginous tissue, osseous tissue, tendinous fibre, vascular fibre, muscular fibre, and nervous fibre.

Cellular tissue (ZELLSTOFF, SCHLEIMSTOFF, *tela cellulosa, mucosa, contextus cellulosus,*) occurs in two states, uniting the parts of the body to each other, and forming the basis of these parts. In the first state, as enveloping or uniting cellular tissue, it is most easily recognised, and appears in the living body as a delicate, semifluid, shapeless, extensible substance, congealing after death, especially when at the same time exposed to the influence of the air or of water, into an irregular flaky texture of fibres and plates. It is denied to be cellular; and our author would rather, with BORDEU, have called it mucous tissue, were it not that under this term has been long understood something quite different; whereas, the universally accepted name of cellular tissue admits of no ambiguity. It is throughout moistened with a watery halitus, and in many places contains fat. In the tropical countries of Asia and Africa, a small thread-like worm, the *filaria medinensis,* is generated in the human cellular membrane; in Europe, one of another description, the *cysticercus cellulosæ,* is met with, and not a winter passes without our author finding them in some dead bodies, and of the same species as exist in monkeys and swine.

The horney tissue *(tela cornea)* is divisible into scales or into fibres. Its cut surface is uniform and smooth, and in thin layers it is transparent; it is hard and elastic, has neither vessels nor nerves; and, being at the same time one of the worst conductors of caloric, is well adapted for covering the more highly-organised parts. It forms, on the one hand, the external envelope *(epidermis)* of the body, with the nails and hair; on the other, the internal membrane *(epithelium)* of the alimentary canal,—perhaps also of the respiratory passages, of the organs of urine and generation, and of the vascular system: nay, all serous membranes seem highly analogous to it. As proofs that the internal lining of the intestinal canal distinctly shows itself here and there as horny substance, are adduced the instances of the gizzards of the Gallinaceæ, and the two first stomachs of the ruminating animals, where the epithelium is evidently of a horny nature; and, in the badger, our author observed the same scaly appearance on the villi of the intestines as is so frequently seen on the epidermis.

The descriptions of the cartilaginous and osseous tissues offer nothing peculiar, or adapted for our analysis.

The tendinous fibre *(fibra tendinea)* is firm and white, and frequently of a silvery lustre. It constitutes, on the one hand, flat bundles of very various form; on the other, membranous expansions: the former are partly connected with muscles as tendons, or they form the numerous ligaments of the body; the latter, with less developed fibres, constitute the perichondrium and periosteum; or, with fibres more strongly developed, the dura mater and aponeuroses; tendinous fibre and cartilage combined, form fibro cartilage.

The vascular fibre *(fibra vasorum)* is principally recognised as arterial fibre *(fibra arterialis)*. It is white, flat, hard, and brittle, and by those characters sufficiently distinguished from muscular fibre; but it likewise exhibits no oscillatory motion during life, and is found to have a different chemical composition, and, when boiled, has quite another taste. As it seems also to be established that arteries, even in warm-blooded animals, are generated anew, another proof is thus afforded of the difference of vascular fibre from muscular fibre, which last, in warm-blooded animals, is never generated or reproduced. This generation of arteries in false membranes, and various tumors, serves to establish a new and evident resemblance between the minute arteries *(arteriellen)* and the fibres of the uterus; which last come and disappear, and, on this account alone, can never be regarded as muscular fibres.

In the description of the muscular fibre, it is stated as being late of forming in the embryo, as being never regenerated in warm-blooded animals, and as never occurring in morbid growths, nor in other parts of the body than where muscles naturally exist.

The nervous fibres *(fibræ nervæ)* are very delicate, very soft, and of a white colour. In the nerves, they are enclosed in very vascular envelopes *(neurilema)*; in the brain, they are without envelopes, and, though always very readily distinguishable in many parts, yet in others they are, as it were, so coalesced as not to be distinguishable, at least not in every condition of the brain. This is still more the case in the spinal marrow and ganglia of the nerves. Examined microscopically, the nervous matter seems to consist of small irregular bodies, usually denominated globules, but which to our author seem much too soft, and not sufficiently separate from each other, to admit of so definite a form being assigned them. The grey substance, which is in many places united to the medullary matter, is composed chiefly, but not entirely, of vessels. The termination of some of the nerves of sense is quite manifest, but every where it seems to be the same,—viz. perfectly distinct: that is to say, a nerve never amalgamates with a vessel, a gland, or the fibre of a muscle; but the nerves surround the vessels, and the smallest muscular fibres, in the form of a network or of a collar. Nervous matter is undoubtedly reproduced in warm-blooded animals, but is not then distinctly fibrous. Nerves are never met with in morbid growths; yet it is not ascertained if the vessels of these growths be without them.

Chapter 2, *of the compound parts.*—The various organs of the body are composed of the preceding simple parts, into which they are all capable of being reduced. Of the compound parts, the vessels and membranes are the simplest, but they again vary very much amongst each other. The vessels are general or particular: to the first belong the arteries, veins, and absorbents; to the latter, the canals of the different secreting organs, the biliary and salivary ducts, the ureters, vasa differentia, &c. They have all at least two coats: those containing blood have three. Longitudinal fibres are easily detected in the veins; but our author has never been able to perceive transverse fibres in the human veins, nor even in the vena cava of the horse; nor has he been able to make out fibres of any description in the thoracic duct, either of man or in that of the horse. The general vessels are widely extended: the absorbents, distinctly such, only occur in the vertebrated animals; vessels containing blood are found in the crustaceæ, arachnoides, molluscæ, and many vermes. Insects (strictly so called) have, in their stead, a peculiar general system of vessels, containing air. The particular vessels extend much further, and are to be detected even in some of the infusoria; for instance, in the vibriones.

The membranes, also, are either general or particular: to the former belong the serous, mucous, and fibrous membranes, the corium, and epidermis (which has been already noticed); to the latter, several coats of the eye, membranes of the brain, &c. In the description of the serous membranes, it is stated that they are throughout void of vessels and nerves, not one of which enters into their structure. Besides those which form shut sacs, as the peritoneum, pleura, pericardium, tunica arachnoidea cerebri, tunica vaginalis testis, the bursæ mucosæ, and the synovial membranes; there are also ranked as serous membranes the internal membrane of all vessels, general or particular; that of the alimentary canal, of the air-passages, and that of the organs of urine and generation. It is denied that any serous membrane itself secretes, but that the fluids transude through them, in the same manner as the perspiration through the epidermis, without any peculiar pores being thereby necessary. They are said also to be equally incapable of inflammation as the epidermis; and that, as in the case of the deposition of various diseased matters on the surface of the body, the epidermis has nothing to do with them; so also, in the case of internal disease, the serous membranes are not concerned. It is only in consequence of disease of the subjacent parts, or of deposition upon their surfaces, that these membranes become changed, thickened, &c.

The mucous membranes *(tunicæ mucosæ, t. propriæ, t. vasculosæ, t. nerveæ,)* have no free side, but are placed between other membranes; are amply supplied with vessels and nerves, and usually are provided with mucous glands *(glandulæ muciparæ).* They stand in the same relation to the skin as the serous membranes do to the epidermis. They cannot, as has been asserted by some, be considered as composed of several layers; they may vary in thickness, but are always single, and the mucous glands, whether superficial or deeper-seated, are placed in them.

The fibrous membranes have been already noticed, in speaking of the simple textures of the body.

The skin, cutis, or corium, the envelope including the whole body immediately under the epidermis, is more dense and compact externally, looser internally, amply provided with vessels and nerves, and in many places with sebaceous glands. It is thicker on some parts of the body than others, as on the face, on the back, or soles of the feet. From its external dense surface appearing so different from its internal loose one, the former being coloured in the Negro,—from effusions taking place in disease between it and the epidermis,—and from the latter falling off and being reproduced, it has been assumed that a peculiar membrane, the rete mucosum of Malpighi, existed between the corium and epidermis; and not only this, but a number of other parts or divisions, have been made out in this situation; all of which are artificial. The seat of the colour of the skin in the Negro is partly in the epidermis, as is readily observed by raising it by the application of a blistering plaster, and partly in the external surface of the corium, which is easily seen to be uniformly black, on soaking the skin of an individual of this stock in boiling water.

The more compound parts are next considered: the glands, the viscera, the connexions of the different parts of the body, and their association into systems, are pointed out. The symmetry which prevails in the human body, and the general preponderance of the right side, are brought under review; whilst allusion is made to the duplicity of many organs, their progressive development, and great uniformity of texture, form, size, and number.

Section III. *General Anthropochemistry.*—Under this head we find the difficulties of animal chemistry pointed out; the unavoidably unsatisfactory nature of its results, in so far as living organised matter is concerned, alluded to; but its great importance, notwithstanding, illustrated,—all organic changes being accompanied with chemical processes. It is only a chemist, who is at the same time a physiologist, who can further and advance animal chemistry; the greatest progress of which is to be looked for from its increasing intimate connexion with physiology and pathology.

Chapter 1, *on the simple elementary substances.*—We are unacquainted with the ultimate elements of animal bodies, which are probably few in number; nor are we justified in assuming the existence of a peculiar animal matter. The elementary substances at present acknowledged as existing in the human body, are oxygen, hydrogen, &c.

Chapter 2, *general organic substances.*—These are gelatine, albumen, &c.; on each of which are made some interesting remarks, the views of Berzelius being, upon the whole, adopted.

Chapter 3, *general compound parts.*—Under this head are ranged the blood, lymph (fluid contained in the absorbents), horny tissue, cartilage, bone, arterial fibre, muscle, and nerve; the composition of each being gone through, the assumption of HALLER with regard to the amount of blood in the human body being from 28 to 30 pounds, is considered not to be exaggerated, in opposition to the opinion of BLUMENBACH, who seems to rate it so low as eight pounds. A number of interesting facts with regard to the globules of the blood, their form, and size, in man as well as in animals, are collected together. The most recent knowledge we possess of the analysis of this fluid, and the other compound parts, is given.

Chapter 4, *on the general chemical processes of the human body.*—It is impossible to regard the materials of our body in any other light than as in a state of multiplied relations or actions upon each other, and these, again, chiefly as chemical processes, or at least as accompanied by such; whether this be in the excretion of some substance or the appropriation of others,—the motion of a muscle, or any other vital action. The general chemical processes give occasion to certain phenomena, of so striking and characteristic a kind, that we are readily induced to attribute them to the agency of peculiar matters, instead of referring them to the universal state of chemical action among the more obvious and tangible elements. These phenomena, or matters (if they must be so called), are heat, light, and electricity. A particular species of heat seems peculiar to all organised bodies, without exception; and we regret that the nature of the subject, and our limits, do not permit us to do more than allude to the number of important facts collected, and judicious observations made on the animal heat of the various classes of animals,—the vermes (Linnæus), the annulata, molluscæ, crustaceæ, fishes, amphibiæ, insects, birds, mammalia, and lastly man. The *source* of heat is attributable in living beings, as well as in unorganised bodies, to alterations in constituent composition. Its *degree* in animals is mainly dependent on the alterations in composition effected by means of respiration; the arguments in favour of which opinion are strongly put. The belief of its being connected with the nervous system is untenable: this system bears no proportion in animals to their degree of heat; for, if this was the case, man ought to have the highest temperature, his nervous system being more developed than that of any other animal; the mammalia should exceed the birds; the latter ought to differ little from the amphibiæ; the insects should rank far below fishes, neither of which are the case. The influence of the nervous system upon the whole animal economy, upon the circulation, and respiration, is so great, that we need not wonder at injuries of it being attended with diminution of the animal heat. The permanent uniformity of the degree of animal heat, under exposure to increased or diminished degrees of temperature, is owing, on the one hand, to the uninterrupted action of the organ on which the production of it depends, and, on the other, to particular accessory means, called into activity in the organismus by those very temperatures.

The luminous or phosphorescent appearance presented by some animals is then noticed, and the electrical properties of others, with a full description of the electrical organs in the torpedo and gymnotus electricus, and the phenomena presented by these animals.

An allusion to the subject of spontaneous combustion concludes the chapter.

Chapter 5, *on the decomposition of the human body.*—With the cessation of life, terminate all those chemical processes in an organised body, whose object is its preservation, and the remains either do not act upon each other, if withdrawn from the atmospheric influence, or, if exposed to this, mostly facilitate their mutual decomposition. The decomposition of organised bodies is characterised by fermentation and putrefaction. The saccharine and acetous fermentations are not confined in their existence to vegetable substances and some animal fluids; for, if the body of a strong healthy man, who has died suddenly, without being poisoned or from preceding hemorrhage, be dissected in mild weather, there is invariably, in a very short time, perceived a disagreeable sweet odour; after this, an acetous smell for a few days; and to this succeeds putrefaction. The rigidity of the body, which is the invariable consequence of death, and antecedent of putrefaction, seems to have attracted a considerable share of attention from our author. With Nysten, he attributes it to the muscles, but not, with that writer, to any remains of muscular power: on the contrary, he connects it with some chemical change taking place in them, from the cessation of the nervous influence.

Putrefaction, and the circumstances which favour or impede it, are then described.

Section IV. *Zoonomy*, takes a general view of life in its most usual and common phenomena, and endeavours to investigate the circumstances and conditions of its existence and continuance, until its final cessation. The term Biology, as expressive of this, is objected to, since it includes too much; more especially since Treviranus has published his great work under this title.

Chapter 1, *on the phenomena of life generally.*—Organismi, or organised bodies, are distinguished from unorganised by consisting of parts which collectively contribute to the preservation or propagation of the whole, by passing through certain stages of development, and exhibiting a periodicity in their actions. All parts of organismi, solid as well as fluid, are endowed with life. A property common to every part of every organismus is excitability or incitability *(incitabilitas)*, by which is meant the power of being urged or roused by stimuli to certain vital actions. These stimuli *(incitamenta)* are external or internal, psychological or physical. The vital phenomena of some parts of the organismus are so little evident, that it is only by comparing these parts in their healthy and diseased state that they are to be discovered. In the soft parts, a common character of the incited state, or of incitement, is a certain fulness or turgescence, which may be augmented or decreased, but never entirely ceases until death. Along with this common character of fulness or turgescence, several systems of organs are likewise distinguished by the possession of a peculiar kind of incitability. In the membranous parts, we call it tonicity—contractility; in the muscles, muscular power—irritability; in the nerves, nervous power—sensibility. From all these is distinguished the intellectual power *(vis psychica)*, yet it ranks next the nervous.

Chapter 2, *on the source or cause of life.*—The cause of life is equally inscrutable as the ultimate principle in physics. The opinions that it is simply chemical, that it is oxygen, or the matter of heat or of electricity, are erroneous; and équally so those which would attribute it to the reciprocal influences of oxygen, carbon, nitrogen, and hydrogen. All these substances are no doubt necessary for life, but of themselves they produce it not, or any thing living; and they are found in the dead remains of living beings. Upon the whole, our author seems to incline to the opinion of REIL, that life arises from the form and composition of matter: the latter can merely be capable of vitality, whilst life itself, or the action of the organismus, first appears along with the form. What composition determines the form of the forthcoming organismus, we are quite ignorant:—we know, however, positively that such only is capable of life which derives its origin from other organismi, an organismus never being formed out of inorganic matter. The expression, *a vital principle,* might be tolerated, if used merely to designate briefly the unknown cause of life; but it becomes most exceptionable, if it be supposed that we thereby explain any thing, or that this vital principle is a something which, being added to the organismus, endows it with life. The designating by individual terms, for the sake of brevity, the peculiar properties or actions of the different systems, need not be objected to; but, independent of the intellectual power, which stands quite alone, it is considered sufficient to assume the following, —viz. incitability, tonicity, irritability, and sensibility, and which are merely manifestations of the same life in different parts.

The opinion that the vital properties are merely modifications of the physical,—that they are merely the latter enhanced *(potenzirt)*, is shown to be untenable; as likewise that which supposes that there is nothing dead in nature, and that there is one universal life, of which each individual life is an efflux. It is impossible to trace a progressive enhancement. What resemblance has elasticity with muscular action? What physical power can be compared with the nervous? Where is the connecting link between the material and intellectual worlds? If the *universal life* is to have a meaning, we must then recur to the old doctrines of emanation, where every thing is an efflux of the godhead; an hypothesis which explains nothing, and stops all inquiry. Finally, the addition of a soul to the body throws no light upon the subject of life. We must, therefore, be contented with considering life as simultaneously given with the organismus, derived from, and propagated by, organismi, without being able to separate and attach it to a particular cause. In a word, we can trace a few of its phenomena, but of its nature we are entirely ignorant.

Chapter 3, *on the different states of life, and their causes.*—Perhaps the beau-ideal of a human being, the most perfect harmony between the utmost possible development, intellectual and physical, has never existed. Even a large development of both in one individual is rare,—usually one preponderates, and frequently both are defective.

Health and disease are then characterised; and the differences of these, according to the temperament, sex, and age, of the individual,—

or according to the climate, mode of life, kinds of food, &c. pointed out and described. In the observations upon climate, we do not find our author attributing so much influence to it as those do who consider all mankind as derived from a single pair. Much more influence is attributed to the originality of the stock, degree of civilisation or mental cultivation, mode of life, habits, food, clothing, &c.

Chapter 4, *on the cessation of life.*—All organised beings bear in themselves the germ of destruction, the mere action of the organs rendering them by degrees more and more unserviceable; and life would sooner terminate, were it not that, during its decay, all the functions are performed with greater tardiness. In those individuals who die simply of senile debility *(marasmus senilis)*, the powers of life are gradually exhausted, and its phenomena sometimes so little apparent, that we almost doubt their existence; as was the case with an individual between eighty and ninety years of age, whom Rudolphi saw for several days before death. Most men, however, and even aged men, die of disease.

Strictly speaking, the time given to sleep should be deducted from our life. Besides this, many animals pass a considerable part of the year in a state of torpor, or rather of suspended animation. The difference between this state of asphyxia in animals, and those instances where men have been overwhelmed and buried for a length of time, and afterwards found alive, is, that the latter have always preserved their consciousness. Having noticed the distinguishing character of suspended animation in man and animals,—viz. the short time the former can remain in this state,—it is observed that we possess very imperfect information with regard to many circumstances attending suspended animation in man; but that they appear in some degree illustrated by observing what takes place during the suspended animation of hybernating animals; a description of the phenomena attending which state is then given. The opinion that cold is the principal agent in producing this state is expressed, and the existence of any peculiar structure or organisation in hybernating animals is denied.

The belief of the existence of life in particular parts after the general life has ceased, in perfect animals, is shown to be erroneous. The absorption and exhalation which take place after death are not vital phenomena; and the supposed growth of the nails and beard after death has arisen from the circumstance of the skin shrinking, and leaving those parts more exposed.

Having now concluded our analysis, we may take the opportunity to observe, that the work is composed in the form of distinct paragraphs, or of aphorisms, which are numbered, and to each of which are annexed annotations or notes. These notes, varying in number and extent, as the matter of the aphorism may seem to demand, are not placed at the bottom of the page, but in the body of it, immediately subsequent to the aphorism to which they belong; a situation to which, by their importance, they are well entitled. The plan itself (at least as here executed) offers considerable advantages in the composition of a Manual, allowing the compression into a small space of much valuable observation, illustration, reference, and criticism, which could not with propriety otherwise find place.

As instances of the manner in which the subjects are treated and the work composed, we shall translate one or two extracts from the original; and, in order to indicate the nature of the errors into which the English translator of the work has fallen, we shall characterise by italics those passages, the sense of which have been misunderstood in the translation; taking care also to give in foot-notes the original along with the faulty translations themselves, that our readers may compare the two together.

The first extract we shall make is from the chapter which treats of the characteristics of the different stocks of men.

§ 63. In the Americans, *the common characters of the skull have not yet been fully made out.** In general the head is small, at least in the South Americans; the forehead low, or slanting backwards. The features are strongly marked, the cheek-bones projecting. The hair is black and coarse, the growth of beard thin and scanty; the colour of the body a bright or dark (copper) red.

*Annot.* 1.—The Americans constitute a number of tribes, which are however nearly allied to each other, and inhabit the whole of America, with the exception of the northern part, occupied by the Eskimaux. (§ 60, B.)

The more northern the region they inhabit, the deeper, upon the whole, is the redness of their skin; yet here also, as in other races, we meet with variations in colour. Frezier (Relation du Voyage de la Mer du Sud, aux Côtes du Chili, &c. —Amst. 1717, 8vo. t. i. p. 121,) *mentions Chilians with fair complexions and a tinge of red in their cheeks, originating, in his opinion, from kidnapped European mothers*:† which is not unlikely. Ye. Ign. Molina (Sagio sulla Storia Naturale del Chili, ba. ii. Bologna 1810. 4to. p. 273,) instances Chilian mountaineers with fair hair and blue eyes; and Felix de Azara (Voyages dans l'Amérique Méridionale, tom. ii. Paris, 1809, 8vo. p. 76,) remarks of the inhabitants of Guaiana, that the colour of their skin is fair, and that some have blue eyes.

*Annot.* 2.—The skulls of the North Americans represented in Blumenbach's Decades, have *little or nothing peculiar in them; more, however, have those, plate 46 of an Aturian,*‡ plates 47 and 48 of Brazilians, and plate 58 of a Botocudan. Caribean skulls (certainly in part deformed by compression during infancy,) are represented in the same work, plates 10 and 20; in Lawrence's Lectures on Physiology, plates 10 and 11; by Hernauld, in the Mém. de l'Acad. des Sciences; and in the Bibliotheque de Planque, tom. iii. p. 646, pl. 72, fig. 1.

*The drawing (on a detached sheet) of the Oneidas, who were, a few years ago, exhibited at Comte's theatre in Paris, is not amiss*,§ but the whole head is not visible; as is also the case with the North-American Indian in Blumenbach's Abbild.

---

* "Ist das Gemeinschaftliche des Schedels noch nicht völlig ausgemittelt."—"The skull, as a whole, is not fully developed." (Howe's *Translation.*)

† "Erwähnt schon Chilesien mit weisser Gesichtsfarbe und etwas Roth auf den Wangen, und leitet diess von den (geraubten) Europäischen müttern ab."—"Informs us that the people of Chili have white complexions, with a tinge of red on their cheeks; and considers this to be on account of the rape of European mothers." (*Ibid.*)

‡ "haben wenig oder nicht Eigenthümliches, desto mehr aber Tab. 46, eines Aturen."—"Nothing in them peculiar, but more particularly that of an Aturian, pl. 46." (*Ibid.*)

§ "Die Abbildung der Oneidas, welche vor ein Paar Jahren auf Comte's Theater in Paris gezeigt wurden, (auf einem eigenen Blatt,) ist nicht üebel."—"The drawings of the Oneidas, which were exhibited a few years ago at Comte's theatre in Paris, (on one sheet,) is not bad." (*Ibid.*)

Nat. Gegenst. plate 2. A Siminole, in Will. Bartram (Reisen durch Nord-und Süd-Karolina ; Berlin, 1793, 8vo. p. 246, taf. 6,) is pretty good. The drawings of Indians of Mehoacan, in Humboldt, (Vue des Cordillères, et Monumens des Peuples d'Amérique ; Paris, 1810; folio ; pl. 52, 53,) *are evidently not portraits.** The figures in the Present State of Peru, (London, 1805 ; 4to. plates 5, 6, 9, 13, 15, 17, 18, 20,) are, for the greater part, embellished. The Prince Max. von Neuwied (Reise nach Brazilien, 1 B. Frankf. a. M. 1820, 4to.) has given drawings of some Indian tribes of the Puris, plates 2 and 3; of the Patachos, plate 7; of the Botocudos, plates 10 and 11, p. 319 but, in these the form of the head has, perhaps, been little attended to. The natives of *Terra del Fuego,*† represented in Sidney Parkinson, (Journal of a Voyage to the South Sea ; London, 1773; 4to. plate 1,) seem to be portraits. Through the kindness of M. Olbers, our anatomical museum possesses two skulls of Brazilian Indians, viz. of the Puri race; of which I shall elsewhere give a more detailed description. They are both skulls of very old individuals, the teeth having fallen out, and the alveolar processes being effaced; notwithstanding which, the greater number of the sutures are still manifest. This, however, sometimes occurs in old individuals amongst us. Moreover, these skulls differ from all others with which I am acquainted, and seem intermediate between the European and the Mongolian. The glabella is broad, and the cheek-bones particularly so, but the latter less so than in the Calmuc ; and the breadth of that part of the upper jaw-bone which contains the teeth is much less. Hence, also, is probably the reason of an incisor tooth in both skulls not having protruded, but remaining within the upper jaw. If this narrowness of the upper jaw-bones, in contradistinction to the great breadth of the cheek-bones, be constant, a very evident character would thereby be established.

Our next extract is from that part of the volume which treats of the Blood.

§ 160. The size of the globules of the human blood I have invariably found to be very small, on frequent examination of that of myself, and also in that of others; and, like Blumenbach, (Inst. Phys. p. 11,) who estimates them at the 3300th, or as Sprengel, (Inst. Phys. i. p. 379,) at the 3000th, I also have found them from the 3000th or 3200th to the 3500th of an inch in diameter; so that a square inch of surface contains nine millions of globules. In fish, I have found them from the 2000th to 2500th of an inch in diameter; so that about four millions would cover the surface of a square inch. In the land salamander, the lesser diameter of the globules is to the greater as seven to ten; seventy of them cover the surface of the tenth part of a square line, and 700,000 that of a square inch ; consequently, the proportion they bear to those of the human blood, is as $12\frac{6}{7}$ to 1. In the same ratio, however, as they increase in size, do they diminish in number; *and, if we take the mass of the globules collectively,*‡ that of man is much larger than that of the animals just mentioned.

*Annot.*—Lar. Spallanzani (De Fenomeni della Circolazione; Modena, 1773; 8vo. p. 210. Experiences sur la Circulation; Paris, an. 8 ; p. 226,) found the size of the globules of the blood in the frog and the tadpole the same, but the number greater in the former. On this point I have no personal experience. *In the red blood of several molluscæ,*|| (Solen Legumen, Tellina nitida, Chama anti-

---

* "Sind wohl keine Portraits."—"Are certainly not worthy of being called portraits." (Howe's *Translation.*)

† "Des Feuerlandes."—"Finnland." (*Ibid.*)

‡ "Und nimmt man die Masse der Blutbläschen zusammen."—"Thus the mass of the blood-vessels collectively." (*Ibid.*)

|| "In dem rothen Blut mehrerer Mollusken-die Blaschen."—"The vesicles in many of the mollusca." (*Ibid.*)—Omitting the "*red blood.*"

quata and calyculata, Arca pilosa, but particularly in the so often examined Arca glycymeris,) Poli (l. o. tab. 2, fig. 1, 5,) found the globules much larger than in man, so that he compares the former to the latter as hemp-seed to millet-seed. I also have found the globules much larger in the punger; and similar observations are met with in Hewson. Whilst examining the blood of the proteus, I did not employ the micrometer, *but the globules appeared** to me to exceed in size all that I had already seen, and at least to equal those of the land salamander. Those of the frog, the lizard, the *tortoise,*† and the common fowl, are at least one-half less, but much larger than those of man, and even those of fishes. Sprengel must either have made a mistake in the number whilst committing his observations to paper, or there is an error of memory, when (Inst. i. p. 379,) he makes the globules of the common fowl as small as those of man : they are as large again, and resemble those of the amphibiæ in form, as Hewson has represented, and Gruithuisen described them. I have been thus circumstantial on this subject, because I believe that, in the variations alluded to, will, at a future period, be found the key to some very important physiological truths. Neither the form nor the size of the globules can be a matter of indifference. What Poli asserts of their turgescence or collapse is interesting, which he considers dependent on the vigorous or enfeebled state of the animal. Micrometrical investigations have their difficulties, but it would be wrong to neglect them on such a subject.

We have thus given a long, but we trust not uninteresting, account of RUDOLPHI's work. Had the translation before us been more accurate. we should have had pleasure in referring to it; but being, as it is, calculated to mislead, we feel ourselves called upon, in justice to our readers, to point out its imperfections. Unwilling as we are to depress the interests of any one who has laboured in the cause of science, we would advise Dr. HOWE to make himself better acquainted with the German language, before he publishes the rest of the work. Nay, there is occasionally an apparent ignorance of some common points in natural history, which makes us rather doubtful with regard to the translator's general qualifications for his task : thus, at page 76, he speaks of the "*spine* of the cuttle-fish," merely because the German expression for the bone of that animal is "der rückenknochen;"—at page 176, the torpedo is said to be "a species of very many varieties," whereas Rudolphi says it is "a genus of very many species;"—and again, "an immense variety has belonged to a former age," for "a gigantic fossil species has belonged to a former age." Instances of this kind might easily be multiplied, but the enumeration would be tedious and unnecessary, because we think we have already said enough to justify the censure we have passed. Finding many mistakes in the Preface, we compared the original with the translation at various parts, (pages 21, 23, 33, 43, 53, 73, 75, 76, 176, 180, &c. &c.;) and it was only after this careful investigation that we determined to express ourselves so strongly.

---

* "allein die Bläschen schienen."—"When the vesicle appeared." (HOWE's *Translation.*)

† "Der Schildkröten."—"Turtle." (*Ibid.*)

Art. III.—*Recherches Anatomico-Pathologiques sur la Phthisie.* Par P. C. A. Louis, Docteur en Medicine des Facutés de Paris et de St. Petersbourg; Membre adjoint de l'Academie Royale de Medicine de Paris, Correspondant de celle de Marseille. *Precedées du Rapport fait al' Academie Royale de Medecine,* par MM. Bourdois, Royur, Collard, and Chomel.—Pp. xxiv. 560. Paris: chez Gabon et Ce. 1825.

*Anatomical and Pathological Researches concerning Phthisis.* By P. Ch. A. Louis, Doctor of Medicine of the Faculties of Paris and St. Petersburg, &c.

Few diseases have exercised the pens of medical writers so often, and to so little purpose, as the one which is the subject of the present article. Although its history and symptoms have been detailed accurately from age to age,—although its pathology has been investigated with a patience and minuteness deserving of a happier result,—still this terror of parents, this fatal destroyer of all that is fair and promising in youth, pursues its destructive course, little (if at all) controlled by the united efforts of the learned of every age and country, and continues to form the most prominent feature in the records of the annual mortality of most of the European nations. Neither can we hope that the exertions of M. Louis will tend to change this melancholy prospect; although, in saying so, we by no means wish to depreciate his labours: on the contrary, we consider that his researches constitute a body of information relative to phthisis, rendering it nearly impossible for any thing to be added to it, and almost perfect, as far as the pathological anatomy of the disease is concerned; but still, with regard to treatment, it offers neither novelty nor improvement; and we moreover think that it affords us too many reasons to conclude that a successful treatment is, in the present state of our knowledge, very little likely to be obtained, and that the most we can expect is to be enabled, in some cases, to keep the malady in check; while the pursuits of business or pleasure, in which mankind is perpetually engaged, forbid us to hope that even this object can be attained in any great number of instances. Let us not be mistaken. We do not reproach M. Louis for not having said more upon the treatment of phthisis; for, in the first place, *that* was not his professed object; and, in the next place, he makes no pretension to any superiority of the kind, and dismisses this part of his subject in nine pages, observing, that he has only to say *un mot sur le traitement.*

M. Louis's work naturally divides itself into two parts: the first containing a minutely accurate account of the diseased appearances of those who died of phthisis,—not only of the contents of the thorax, but of every organ of the body. The second part describes the symptoms of the disease, and is, as well as the first portion of the work, interspersed with many highly interesting cases, illustrating the latent, the chronic, and acute phthisis; together with the various modes in which the fatal termination takes place.

A pretty long advertisement (twenty-four pages) ushers in the work, in which the author explains his design in undertaking it. "The cases which served as a basis to our researches, says M. Louis, were taken at the Hospital la Charité, reckoning from the three last months of the year 1821: from that epoch, the history of all the patients admitted into the wards of M. CHOMEL have been collected, comprising forty-eight beds, equally divided between male and female patients."

In order to do complete justice to this undertaking, we find that our author renounced private practice to pursue this inquiry, and that he passed three, four, and sometimes five, hours daily in the hospital, never bestowing less than two hours upon each anatomical investigation. The total number of cases, the result of which is given, amounts to 123, of which fifty are recorded in the work itself. It is to be observed, however, that, during the three years which our author has consecrated to this labour, the number of patients has amounted to 1960: of these, 358 have died; 127 of this number died of phthisis, and forty others, who perished of different diseases, had also tubercles in the lungs. Such are the strong claims to our attention which M. Louis possesses.

We now proceed to consider the contents of the first part of the volume, premising that we cannot attempt a copious analysis of a work extending to nearly 600 pages, but that we shall endeavour principally to mark those points of interest which have struck us in the perusal.

Our author commences by confirming the opinion of LAENNEC, that the existence of tubercles in the lungs is the cause, and constitutes the essential character, of phthisis; and he therefore rejects the division of this disease into tubercular, granular, cancerous, melanose, calculous, and ulcerous, as attempted by BAYLE. After describing the appearance of tubercles, with which we are unfortunately too familiar, he observes that they are almost always largest, most numerous, and more advanced in progress, towards the upper part of the lungs. The tubercles were accompanied with productions of a very different character, small round bodies of a homogeneous structure, shining, and of considerable hardness, from the size of a small pea to that of a grain of millet, which are called granulations. These constitute, as M. Laennec has observed, the first rudiment of tubercles; and these are also, as well as the tubercles themselves, larger and more numerous in the upper part of the lungs. At a certain period of their existence, they presented an opaque yellow spot in their centre, and, the higher they were situated, the larger was this spot. Most frequently they were found at a certain distance from the pleura, but in about a third of the subjects this was not the case; in one instance only they existed on the superficies of the lungs. Though it is not easy to define the period necessary for these granulations to acquire the size of a pea, in some cases their development is very rapid, not occupying more than three or four weeks; in others, years appeared necessary to produce this effect.

This greyish, semi-transparent matter sometimes was found in other forms, in irregular masses; and it was sometimes found, though rarely, in other organs of the body. A certain quantity of this matter was always to be found around tubercular excavations of any considerable size.

Another kind of substance was also sometimes met with in the lungs of those who died of phthisis, described also by Laennec: it was of a consistence less firm than the granulations, and more transparent, reddish, though sometimes pale and having the appearance of jelly.

Most commonly tubercles were found in both lungs; but in five instances they were confined to the left side, and twice only to the right. The softening of the tubercles took place at very different periods: in some cases, from the twentieth to the fortieth day from the commencement of the malady, though commonly it required a longer time. It followed the same progress as the transformation of the grey matter into tuberculous matter, beginning in the centre of the tumor, and descending from the upper part to the base of the lungs. Sometimes, however, instead of being successive, this change took place simultaneously throughout a considerable extent. Such instances were confined entirely to the acute phthisis.

The cavities of tubercles have been found entirely empty before the end of the third month: at this period their parietes are usually soft, and lined with a false membrane, which is easily removed. When the malady was of an older date, their parietes were more or less hard, and combined with other circumstances of less importance; but, whether large or small, recent or of long standing, these excavations communicated with the bronchiæ by a greater or less number of openings; the mucous membrane of the one, and the false membrane of the other, being intimately united at the opening. The matter contained in these excavations differed in many respects, especially in regard to the date of their existence. When recent, it was thick and yellowish, similar to common pus; when of long standing, the liquid was greenish or yellowish, of a dirty disagreeable aspect, of a middling consistence, sometimes marked with blood, at others very red, though generally without odour. The matter from these excavations had sometimes the smell of animal substances which have been macerated for some time, and this odour was independent of the size of the cavities.

Such are the principal remarks made by M. Louis relative to the lungs.

Two curious cases are subjoined; in one of which a large excavation was filled by a fibrous substance, instead of pus; the other presented the remarkable circumstance of the existence of a fragment of the pulmonary tissue, perfectly healthy in appearance, enclosed in a cavity, without adhering to any part of its parietes.

*Pleura.*—Adhesions of the lungs to the pleura were the most common appearance; so much so, that, out of 112 instances, only one was found in which both lungs were perfectly free from them. A proportion was also always observed between the adhesions and the internal malady. An extravasation of a clear serous fluid, to the extent of about a pint, was also met with several times in the cavities of the pleura. This extravasation, which occurred in about a tenth of the patients, sometimes came on very suddenly. The same effusion was, however, found to take place at the termination of other chronic affections; and, excepting in diseases of the heart, in about a fourth part of these cases. This seems to indicate that this species of hydrothorax is really independent of the original malady.

*The Epiglottis, Larynx, and Trachea.*—M. Louis remarks, that pathologists have been in the habit of confining their observations principally to ulcerations of the larynx, whilst those of the epiglottis and trachea have escaped their notice. Ulcers of the epiglottis are, however, scarcely more rare than those of the larynx, bearing the proportion of eighteen, twenty-two, and thirty-one, to similar affections of the larynx and trachea, out of two hundred subjects carefully examined.

When the trachea is ulcerated, the mucous membrane is of a vivid red colour; though occasionally, when the ulcerations are numerous, but small, it preserves its white appearance: it is in the lower half of the trachea that the redness is most marked. When small, these ulcerations were pretty equally distributed throughout the canal, and vice versâ; their character was otherwise the same. In some subjects, a certain number of cartilaginous rings were denuded, and sometimes destroyed in part. Our author thinks that the situation of large ulcerations at the posterior part of the trachea, arises from the habitual passage and residence of the expectoration at that part. In the third part of the cases, where this canal was not ulcerated, the mucous membrane was of a red colour, increasing in depth towards the bifurcation. It was more intense also towards the fleshy portion, and depended, most probably, upon the same causes as the ulcerations.

Ulcers of the larynx were not quite so common as the above, and showed themselves seldom unless as accompanying them: their seat was most commonly at the union of the chordæ vocales, one or other of which was frequently destroyed.

Ulcers of the epiglottis were met with in a sixth of the cases, and five times only without complication with those of the larynx and trachea. The ulcers of the epiglottis were frequently of some depth, but the mucous membrane did not appear to be much thickened. The laryngeal surface was always affected, and generally the lower portion only.

No instance occurred of tubercular granulations being found either on the surface or in the substance of the epiglottis, larynx, and trachea; so that inflammation solely may be considered as their cause. These ulcerations were also much less common in women than in men, in the proportion of one to two.

Our author's remarks upon the condition of the *Circulating System* will not detain us long. The heart presented no remarkable alterations in structure; the pericardium, as might be supposed, exhibited some traces of inflammation in a few instances, and a few ounces of serosity were found in its cavity in about one-tenth of the cases; so that, as far as phthisis is concerned, the appearance of this organ did not materially differ from those often met with at the termination of other chronic complaints. Redness of the internal and middle tunics of the aorta was found in about one-fourth of the subjects examined, and these principally in individuals from the twentieth to the thirty-first year of their age. Other organic alterations of the coats of the artery were also observed, but in a proportion different from that usually met with in other diseases. The yellow, white, or cartilaginous spots were only observed after a certain period of life, from thirty-six to forty; and then they existed, in a greater or less number, in all the individuals examined. Their presence was not denoted by any particular symptom; and our author doubts their commonly asserted origin from inflammation.

*Pharynx and Œsophagus.*—The lesions of these parts are not frequent. Out of eighty subjects, the pharynx was only twice found to be affected with ulcerations, which were numerous, small, and equally distributed throughout its whole extent. Ulcerations of the œsophagus were also found exactly in the same proportions. The internal surface of this canal was sometimes covered with a kind of false membrane: in this case, the epidermis of the œsophagus had disappeared, but the mucous membrane remained quite sound. There did not appear to be any symptoms indicating the presence of these diseased conditions: nevertheless, the bodies of individuals, who died of any other disease excepting phthisis, did not present either ulceration or softening and thinning of these parts.

*Stomach.*—Several remarkable changes were observed in this viscus, both with respect to its volume and position. In nine cases out of ninety-six, the stomach was three times its natural size, and of course descended below its natural position. These changes were peculiar to phthisical patients; and our author conjectures that incessant coughing may be the cause. Independently of this increase of size, the mucous membrane of the stomach was softened, and had become thinner, in several subjects; it was even destroyed in some, and of a red colour, of a greater or less degree of intensity. This softening of the stomach existed in a fifth of the bodies examined. The parts wherein it was most observable were of a bluish or yellowish cast, and remarkable for the number of large vessels, sometimes filled with black blood. The mucous membrane was white, and semi-transparent; it was extremely soft, so as occasionally to have scarcely more consistence than moderately thick mucus: under this, the cellular tissue was commonly healthy. It would appear, from other observations, that the above conditions of the mucous membrane are the result of inflammation. Neither, says M. Louis, does the discoloration and thinning of this membrane contradict this assumption; for the long continued application of a blister will produce the same effects on the skin. The above lesion was more frequently met with in women than in men.

Redness, united with a thickening and tuberculated appearance, was sometimes observed; and these appearances our author considers to be most decidedly the effects of inflammation.

Ulcerations of the mucous membrane occurred in a twelfth of the cases: they were usually small, not numerous, and complicated with some other diseased appearance of the part. Other deviations from the healthy state are also mentioned as occasionally remarked,—such as general redness of the whole membrane, disappearing after a maceration of two or three hours; general softening, without any alteration either of colour or thickness; in one case, an appearance of cicatrization of the membrane, and in another the conversion of the muscular coat, for a small extent, into a cartilaginous substance. It is to be remarked, that these alterations of structure are not peculiar to phthisis, but they are met with, in very different proportions, in subjects dying of other diseases.

The duodenum was almost always found in a natural state. In three cases only were there ulcerations; in a few others, the mucous membrane was reddish, and sometimes of a grey colour, which appeared to arise from an infinite number of black points, with which it appeared to be sprinkled. Frequently the mucous follicles were observed to be increased greatly in size, but without any alteration of structure. But these appearances were equally remarkable in patients who died of any other chronic disease.

Our readers will perceive that M. Louis treats of the duodenum by itself, restricting the term *small intestines* to the jejunum and ileum.

After devoting a page or two to a description of the healthy appearance of the mucous membrane of these intestines, our author observes, that the elliptic patches formed by the agglomeration of mucous cryptæ, and which are met with in the natural state of these parts, participate very little in the lesions of the mucous membrane that surrounds them, and are often the seat of disease which that membrane does not partake of. These patches were the most ordinary seat of ulcerations in those who died of fever, as well as of phthisis: they were met with in five-sixths of the phthisical subjects. When they were confined to the mucous surface, the cellular tissue was very much thickened; and, when that was destroyed, the muscular coat was equally thickened; so that, in proportion as one membrane was destroyed, the next had become thicker. The mucous membrane was sometimes also redder than natural, but it was seldom softer or more dense.

The granulations met with in the small intestines were of two kinds; sometimes resembling the tubercular matter, at others whiter and harder, almost cartilaginous. These last (M. Louis calls them semi-cartilaginous,) were very numerous, existed the whole length of the intestine, their number and size increasing towards the cœcum; and they were met with from the size of a small pin's head to that of a pea. These granulations were occasionally situated upon the patches above mentioned, but most commonly in the intervals between them. The tubercular granulations were not found in any instance so numerous as the above; and to these succeeded small ulcerations, the mechanism of which was the same as that of the tubercular excavations met with in the lungs.

Ulcerations were still more common, and therefore appeared to be often independent of either species of granulations. The dimensions of these ulcerations varied from one line to five or six inches; they were largest and most numerous towards the cœcum. They were variable also in form: when small they were round, elliptical when larger; and the linear form was the most rare. They did not differ less in colour. Their structure also was different according to their size; and they frequently seemed to be produced by the re-union of several small ones.

Besides the above-described lesions, small abscesses, of the size of a pea, were occasionally found; and these existed where there were neither ulcerations nor tubercular granulations. These abscesses have scarcely ever been observed but in phthisical subjects. When the small intestines were sound, or nearly so, they contained a greater or less quantity of mucus, of various colour and consistence, sometimes streaked with blood; but, when ulcerations were large and numerous, instead of mucus there was a fluid, more or less thick, of a reddish colour, and of a strong odour.

Many of the above appearances are common to other diseases besides phthisis, but the tubercular granulations and the ulcerations appear to belong solely to this; though these latter have certainly been found, very rarely, in other cases.

Excepting the semi-cartilaginous granulations, the morbid appearances of the *large intestines* were similar to those of the smaller ones. We may remark, however, that the softening of the mucous membrane appears to be the effect of inflammation, coming on during the last days of the patient's life.

The *lymphatic glands* were frequently tuberculous, sometimes more or less red and increased in size. Those which presented most frequently the tuberculated appearance were the following, in the order in which they stand,—viz. the glands of the mesentery, the mæso-cœcum, the mæso-colon, the neck, the loins, and axilla. With respect to the bronchial glands, M. Louis affirms that they are not oftener tuberculous than those of the mesentery. Of these latter glands, all were not equally subject to this affection: those nearest the cœcum were generally the most diseased. They were seldom met with in a softened condition; owing, most probably, to their recent date. The mesenteric glands, when tuberculous, had acquired a more considerable size than ordinary: when they exhibited a small number of tuberculous points, their tissue was of a colour more or less red, and they were often somewhat softened. But in some cases these glands, although they had acquired some increase of size, had not sensibly changed either their colour or consistence; so that if, on one side, inflammation appeared to have had some influence in the development of the tuberculous matter, on the other it appeared, in many cases, to have had no share in it. M. Louis asks, whether the appearance of these tubercles had constantly any reference to the inflammatory condition of the mucous membrane to which they corresponded? In every case, he observes, in which these glands were tuberculous, ulcerations of the small intestines were found; and these ulcers had not taken place without a primitive or consecutive inflammation of the mucous membrane: they were of themselves, indeed, a perpetual source of irritation.

The glands of the mæso-colon and the loins were less frequently tuberculous than those of the mesentery. The lumbar glands were found tuberculous in only five cases out of sixty.

The cervical glands were more or less tubercular in the tenth part of the subjects; and in one-half the number the mucous membrane was redder than natural. In one case only the cervical glands, changed into tuberculous matter, gave rise to pain; and this patient furnished the only example of tubercles developed in the axillary glands.

When the bronchial glands were tubercular, they were generally of a large size, and of a grey or blackish colour.

These morbid appearances seem to be peculiar to phthisis: out of ninety-eight individuals, who died of other chronic diseases, no instance of tuberculous lymphatic glands was met with, though inflammation of the mucous membrane of the intestinal canal, as well as ulceration, was frequently observed; which forms an additional ground for believing that inflammation is not the only, nor the most important, agent in their transformation.

The *liver* presented, in about one-third of the subjects, the appearance of a fatty transformation. In this condition, the liver was pale, of a fawn colour more or less deep, spotted with red both within and without. It preserved its shape, but was almost always increased, sometimes even to double its natural size. The great lobe was usually the seat of this alteration; and then the liver covered a great portion of the anterior face of the stomach, extended two or three fingers' breadth below the false ribs, and occupied the epigastric region. In one case it was found in the middle of the belly, and two inches distant from the pubis. In texture it was usually softer than natural. When the alteration was more advanced, it greased the knife and the hands like common fat.

The causes of this alteration appeared to our author obscure; but it is remarked, that it is almost peculiar to phthisical subjects. Sex also appears to have some influence in producing it, having been observed only in ten male subjects out of forty-nine. Neither the strength nor weakness of the constitution, nor the age of the patient, seem to have any connexion with its production.

This condition of the liver may be produced very quickly. It was observed in one case where the whole of the symptoms of phthisis ran their course in fifty days; though the rapidity or slowness of the original disease did not influence the proportion of fatty transformations in the liver in other cases. M. Louis knows no symptoms that particularly indicate this state; and therefore there is only one circumstance, the increase of size, that can lead to the suspicion of its existence. Whenever it was met with, no other organic lesion of the liver existed in conjunction with it. Indeed, these were very rare; twice only was any quantity of tuberculous matter found in it. In another subject, a woman of twenty-nine years of age, the middle lobe was destroyed, and a fibrous cyst occupied its place, nearly round, and twice the size of the part which it replaced.

In the *gallbladder* no appearances, that would seem to be exclusively belonging to phthisical subjects, were found.

*The Spleen.*—If, says M. Louis, the ignorance with regard to the uses of this viscus renders the study of its morbid appearances less interesting than that of other organs, their number and frequency ought to excite the zeal of observers; and therefore he has thought it right to enumerate those which he has met with. These alterations regarded the consistence and volume of the organ, and the development of accidental tissues: these were of two kinds; one, the tubercular, which was observed in seven out of ninety bodies, carefully examined. The tubercles were very numerous; they were not encysted, and the viscus round them was healthy: in no instance was the grey tubercular matter seen by the side of them. The second kind consisted of round, yellowish, shining granulations, elastic and humid, and very different from the tubercular matter. They were placed in the midst of the spleen, which was softer and larger than natural. These granulations were met with in only one case. The volume of the spleen was diminished in fifteen subjects; and increased to double, triple, or even quadruple, in sixteen. M. Louis could not connect this increased size with any previous affection of intermittent or continued fever. It differed no less in consistence than in size. Excepting in the absence of tubercles, the other diseased conditions of the spleen do not appear to be peculiar to phthisis.

The *urinary organs* seldom presented any remarkable diseased appearance. In the renal capsules, a small quantity of tuberculous matter was observed in two cases. The kidneys were in all respects perfectly sound in three-fourths of the subjects. In three cases only they exhibited a certain quantity of tuberculous matter; and in one case this extended to the corresponding ureter. The rarity of these appearances induces M. Louis to relate the case in which they were observed. We present our readers with a translation of it.

"A hair-dresser, twenty-four years of age, scrofulous, and of a delicate constitution, born of healthy parents, suffered a severe sprain of the right foot at twelve years old. After having experienced violent pains for two years, they ceased, and only re-appeared occasionally. Some fistulous openings established about the ankle-joint, afforded, almost without interruption during four years, a certain quantity of pus. The patient had, however, not discontinued his occupations, and often went considerable distances without inconvenience. He coughed and expectorated for nineteen months prior to his admission into La Charité, which was on the 16th February, 1822. When the cough first began, severe pain in the right side of the chest had required the application of a great number of leeches opposite to the site of the pain; he had not experienced hæmoptysis, and the difficulty of breathing only existed for six months. His appetite had fallen off for more than a year, and within the last four months had entirely forsaken him; the thirst was intense, and the diarrhœa frequent. He did not recollect to have been troubled with sweats, but had suffered from shivering fits for fifteen days.

On the 17th February, his emaciation extreme, with considerable debility. He has neither headache, nor pain in the loins or limbs. His intellects sound; his speech short; the voice clear; respiration accelerated; cough rather frequent, sometimes in fits; expectoration not abundant, greenish, and opaque. A clear sound on percussion generally throughout the chest, excepting between the shoulders and under the right axilla. The skin hot, heat violent in the evening; no sweats nor chills during the preceding night; the pulse moderately accelerated. The tongue clean, thirst great, appetite nearly gone; nausea occasionally after coughing. Four liquid evacuations, with flatulence and colicy pains."

The following days, the diarrhœa was more severe, but the other symptoms remained unchanged. He was a little deaf from the 12th to the 15th of May, the day of his death, which took place after twenty-four hours of delirium.

On examining the body, nothing particular was observable as to its external appearance. (The brain was removed for anatomical investigation.) In the chest, the lungs adhered to the pleura costalis, at the upper and posterior part. At the top of the left lung, there was a small tubercular excavation; and, throughout the remainder of its extent, a good many grey, semi-transparent granulations, round which the pulmonary tissue was quite sound. On making an incision from the summit to the base of this organ, a great number of round openings were seen, which were nothing more than the orifices of the bronchiæ, more or less thickened, and uniformly dilated, almost to the very surface of the lung. The same lesions existed on the right side, but the granulations were more numerous, and the bronchiæ still more dilated. The heart was of a middling size. In the abdomen, the mucous membrane of the stomach was red around the cordia. The small intestines presented some ulcerations; some very large ones were found in the cœcum. The colon, and the other viscera, excepting the right kidney, were in a healthy state. This kidney was of the natural size, and its position was not changed; but it was yellowish in the upper third of its extent. The corresponding ureter was hard, was four lines in diameter, and was less dense and smaller as it approached the bladder. The tissue of the kidney was destroyed in the upper third, and replaced by a yellowish opaque matter, really tuberculous, which rested upon a false membrane of the same nature. This was prolonged downwards, lined the pelvis of the kidney and the ureter, to the parietes of which it was firmly affixed. Though firm on that side, it became soft and friable in proportion as it approached its outer surface, it had from half a line to a line of thickness, and was more consistent in the ureter than elsewhere. By accident, the bladder was not examined; but our author observes, that this viscus, though more or less distended in many cases, afforded no other diseased appearance; and with respect to the kidney, he adds, that, in two hundred subjects examined, who had died of other diseases, not a single portion of tuberculous matter was found in any one.

Respecting the *male genital organs*, the only remark we shall extract is that, out of forty subjects, in which the prostate gland, the vesiculæ seminales, and the vasa deferentia, were carefully examined, three presented a greater or less quantity of tuberculous matter in the prostate; and in one, whose case is related, this matter existed in all these several parts. Another fact of some importance is here incidentally mentioned by our author. He says that, wherever the tubercular matter was found to exist out of the lungs, whether in the spinal marrow, the mesentery, spleen, or prostate, &c. it was always developed in an equal degree, not having become softened; which would seem to indicate that one and the same cause was operating at the same time upon all these points.

The *female organs* of generation offer no point of peculiar interest to arrest our attention. The uterus is said generally to have been smaller than natural. Once a small quantity of tubercular matter was found in this viscus, and twice a little was perceived in the ovaria.

In the *peritoneum*, an effusion of serum was found in many cases,—that is to say, in a fifth part of the subjects,—in the quantity of from one to eight pints. Men were affected in the same proportion with women; and it was not more frequent where the liver and mesentery were diseased, than in the contrary state. Sometimes, together with the serum, there was either a certain quantity of thick pus, or a yellowish, soft kind of false membrane. This was certainly the result of acute peritonitis, and was only met with four times, and had taken place only a few days before death. Ancient adhesions were found in three subjects. In one case, a number of miliary granulations existed on the free surface of the peritoneum, placed in the midst of a false membrane, almost opaque; and lastly, in another remarkable case, the same matter was found in the great epiploon, and in the meso-colon.

*Of the brain, and its membranes.*—The lesions of these parts observed by our author, which are peculiar to phthisis, are hydatids and tubercles; though he afterwards qualifies this assertion respecting hydatids. The following case is interesting:—

"A stone-cutter, forty-four years of age, of a hardy constitution, sober, industrious, and seldom subject to illness, became affected, within the last three years, with a complaint in the throat, lasting from twenty-four to thirty-six hours; and with a diarrhœa, which had lasted still longer, and which came on from month to month, lasting a day or two, but without pain. Six months prior to his admission into the hospital, he was seized on a sudden, without any known cause, and without cough, with a vomiting of blood, which he supposed amounted to two pints; and, some days after, he passed a considerable quantity by stool. In consequence of these losses of blood, he had kept his bed for several days, and had not worked for three months. Cough and expectoration commenced from the time of the vomiting of blood; cold chills, followed by heats and sweating, had become established for the last two months, and the respiration had been affected for the same length of time; the appetite had diminished, but the thirst was considerable. He had neither experienced pain nor diarrhœa.

The 26th November, 1822, the day after his admission into the hospital, he appeared rather feeble, without headache; intellects clear, respiration tranquil; pectoriloquism imperfect between the vertebral column and scapulæ; respiration hard and laborious in the same points, but natural elsewhere. Cough not very frequent, expectoration round and in balls, opaque, united by a viscid phlegm. Some pricking pains in the lateral parts of the chest. A hoarse, broken voice; a feeling as if the larynx was torn, whilst coughing or swallowing. The heat natural; pulse quiet, regular, and under seventy pulsations in the minute; very little thirst, deglutition imperfect, although the pharynx and tonsils appeared natural; the belly indolent.*

The following month, the state of the patient appeared to be somewhat ameliorated. He felt no cold chills; nevertheless, at times the aphonia was complete. The appetite also mended.

From the 24th of December to the 31st of January, the patient preserved his intellectual faculties; slept but little; had no headache; and lost his strength slowly. The aphonia was variable. A constant pain about the thyroid cartilage, accompanied with heat, especially in the night. Deglutition of the saliva troublesome. The cough and oppression increased during the last ten days of the month of January; but were a little less after the 15th, and the expectoration became thicker. At the same time there was a severe pain on a level with the left breast, but without any alteration in the sound of that part. The respiration was tracheal, accompanied by a gurgling noise *(gorgouillement)* under the left clavicle, for the extent of five inches: it was the same in the corresponding point behind, but of a less considerable extent. There was also a little mucous rattle on the right side. The pulse continued to be as slow as usual; the chills appeared again in the evening, followed by heat and sweat.

From the 26th to the 28th December, violent colics took place, followed by dirrrhœa, and which continued more or less throughout the month of January. The appetite failed, from the appearance of the diarrhœa.

On the 31st, the weakness suddenly became considerable; his face very pale. He complained of a very troublesome feeling of weakness at the epigastrium; the breast gave no sound under the left clavicle, for the extent of three inches. The expectorations resembled a greyish and greenish pus, were marked with red. The pulse was calm and regular. There was some delirium in the night; and at three o'clock the next morning the patient died.

*Examination of the body, twenty-nine hours after death.*—Nothing remarkable in the external condition. In the head, the dura mater adhered very firmly to the sagittal suture; there was no infiltration under the arachnoid. At the upper part and sides of the brain, below the pia mater, about twenty vesicles were seen, that extended beyond the convolutions about a line or a line and a half; the rest was buried in the cerebral substance, which was perfectly healthy around them. These vesicles were of a round form, and of various dimensions: three of them were the size of a nut, uniform on the surface, and presented a kind of pedicle, from which a white and opaque membrane arose, and which did not cover the hydatid throughout its whole extent. The brain was very vascular; the lateral ventricles, the cerebellum, &c. in a healthy state.—In the neck, the mucous membrane of the laryngeal face of the epiglottis was destroyed the whole of its height; the borders of the ulceration were a little thickened, hard, and white; its base was unequal, and of a reddish colour. Two small ulcerations above the chordæ vocales: that of the right side was nearly destroyed; the circumference

* We omit the treatment, as wholly unworthy of insertion.—Editors.

greyish, hard, and lardaceous. The mucous membrane of the trachea red, and a little thickened inferiorly; that of the bronchiæ still more red; both, however, without ulcerations.—In the chest, on the left side, rather less than a pint of reddish serum, in the midst of a false membrane which lined the lung. The diaphragmatic and costal pleuræ were of a deep red, and were half a line in thickness. At the top of the upper lobe of the lungs was found a large excavation, lined with a semi-cartilaginous false membrane. On the sound lungs were some tubercles, or little masses of melanosis: throughout the rest of its extent there were many little cavities, for the most part but incompletely emptied. The inferior lobe contained a considerable number of grey granulations, without tubercular matter or excavations. On the right side the lesions were the same, though not quite to so great an extent. The aorta, below the origin of the cœliac artery, presented some cartilaginous and bony patches. The mucous membrane of the stomach was red in some spots, and a little softened at the great curvature. On the left of the cardia there was an ulcer, half an inch in diameter, the borders of which were irregular. The mucous membrane of the small intestines was perfectly sound, excepting two small ulcerations, which presented some semi-transparent miliary tubercles on their surface. The mucous membrane of the large intestines was as soft as mucus, and of a violet red in several points in the rectum; there were ten small sub-mucous abscesses, of the size of a pea, and eight ulcerations, of the same size. The spleen was also softened."

Excepting the hydatids, all the other lesions of the above case had given rise to symptoms proper to each of them.

The relation of another long and interesting case next follows; the most remarkable circumstance attending which was the simultaneous existence of tubercles in a number of different parts,—the lungs, neck, axilla, mesentery, loins, and spleen; and especially the equal state of their development in all these parts, excepting the lungs.

M. Louis finishes the pathological part of his work with a recapitulation of the principal facts recorded therein.

