# Errata in Our Last Number

**Published:** 1826-04

**Authors:** 


ERRATA *in our last Number.*

Page 223, for Phthiscologia, read Phthisiologia.
— 224, for *engaged* extensive opportunities, read *enjoyed*, &c.
— 228, for *these* morbid *alterations*, read *those* morbid *operations*.

